# Complete versus simplified Selvester QRS score for infarct severity assessment in ST-elevation myocardial infarction

**DOI:** 10.1186/s12872-019-1230-0

**Published:** 2019-12-09

**Authors:** Christina Tiller, Martin Reindl, Sebastian Johannes Reinstadler, Magdalena Holzknecht, Michael Schreinlechner, Alexander Peherstorfer, Nicolas Hein, Ivan Lechner, Agnes Mayr, Gert Klug, Bernhard Metzler

**Affiliations:** 1 Cardiology and Angiology, University Clinic of Internal Medicine III, Medical University of Innsbruck, Anichstrasse 35, A-6020 Innsbruck, Austria; 2 University Clinic of Radiology, Medical University of Innsbruck, Anichstrasse 35, A-6020 Innsbruck, Austria

**Keywords:** ST-segment elevation myocardial infarction, Electrocardiography, Cardiac magnetic resonance imaging, Risk stratification

## Abstract

**Background:**

Complete and simplified Selvester QRS score have been proposed as valuable clinical tool for estimating myocardial damage in patients with ST-elevation myocardial infarction (STEMI). We sought to comprehensively compare both scoring systems for the prediction of myocardial and microvascular injury assessed by cardiac magnetic resonance (CMR) imaging in patients with acute STEMI.

**Methods:**

In this prospective observational study, 201 revascularized STEMI patients were included. Electrocardiography was conducted at a median of 2 (interquartile range 1–4) days after the index event to evaluate the complete and simplified QRS scores. CMR was performed within 1 week and 4 months thereafter to determine acute and chronic infarct size (IS) as well as microvascular obstruction (MVO).

**Results:**

Complete and simplified QRS score showed comparable predictive value for acute (area under the curve (AUC) = 0.64 vs. 0.67) and chronic IS (AUC = 0.63 vs. 0.68) as well as for MVO (AUC = 0.64 vs. 0.66). Peak high sensitivity cardiac troponin T (hs-cTnT) showed an AUC of 0.88 for acute IS and 0.91 for chronic IS, respectively. For the prediction of MVO, peak hs-cTnT represented an AUC of 0.81.

**Conclusions:**

In reperfused STEMI, complete and simplified QRS score displayed comparable value for the prediction of acute and chronic myocardial as well as microvascular damage. However, both QRS scoring systems provided inferior predictive validity, compared to peak hs-cTnT, the clinical reference method for IS estimation.

## Background

In survivors of acute ST-elevation myocardial infarction (STEMI), the severity of myocardial damage is of major prognostic relevance [1]. Cardiac magnetic resonance (CMR) imaging enables a precise and comprehensive assessment of infarct severity after STEMI [2], but is still limited due to restricted availability in daily routine.

Previous studies have documented the capability of different electrocardiographic (ECG) markers for prognosis assessment after STEMI [3]. The Selvester QRS Score has been proposed as indicator of infarct size [4]. However, the original Selvester QRS score (=“complete QRS score”) is very complex compromising 54 individual criteria. Accordingly, a simplified version (=“simplified QRS score”) has been formed including only 37 criteria [5].

Both QRS scoring systems have been shown to correlate with the extent of myocardial damage [6, 7]. However, to date no study has directly compared the value of complete versus simplified QRS scoring system for the estimation of myocardial and microvascular damage.

The present investigation aimed to evaluate a direct comparison of complete and simplified QRS score as well as in comparison with the clinical reference method peak hs-cTnT for the prediction of CMR-derived myocardial damage in a cohort of STEMI patients treated with primary percutaneous coronary intervention (pPCI).

## Methods

### Study design

**Fig. 1:**
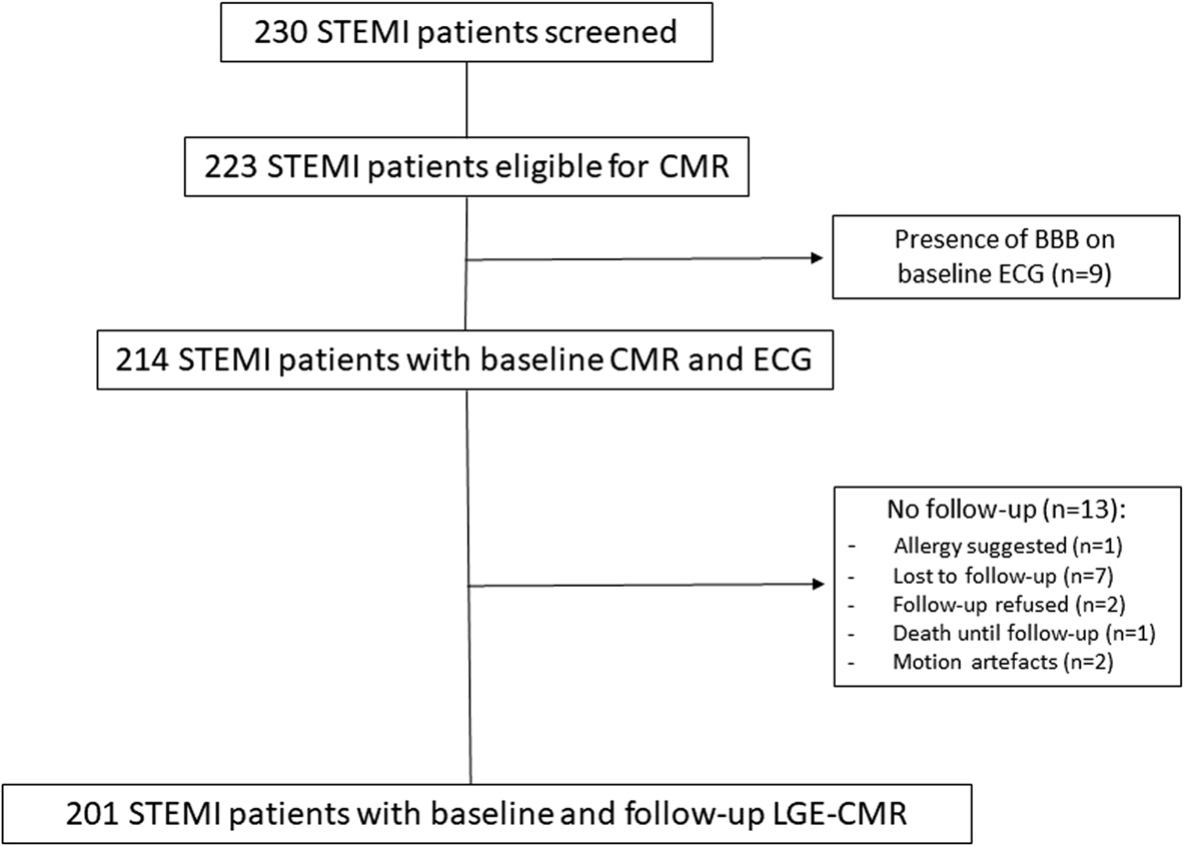
Flow diagram of the present cohort study. Abbreviations: STEMI=ST-elevation myocardial infarction; CMR = Cardiac magnetic resonance; BBB=Bundle branch block; ECG = Electrocardiogram; LGE = Late gadolinium enhancement

In this single-centre prospective observational study, we included 201 STEMI patients admitted to the coronary care unit of Innsbruck University Hospital. The flow chart of the present study is presented in Fig. 1. Inclusion criteria were first-time STEMI diagnosed in accordance with the redefined ESC/ACC committee criteria [8] and revascularization by pPCI within 12 h after symptom onset. Exclusion criteria were age under 18 years, any history of prior myocardial infarction or coronary intervention, an estimated glomerular filtration rate < 30 ml/min/1.73 m^2^, Killip class > II and any contraindication to CMR examination (pacemaker, aneurysm clips, orbital foreign body, claustrophobia, known or suggested contrast agent allergy to gadolinium). Furthermore, patients with bundle branch or fascicular block were excluded.

A standard 12-lead surface ECG (voltage: 10 mm/mV; speed: 25 mm/s) was conducted at discharge, at a median of 2 days (Interquartile range [IQR 1–4] after STEMI. Complete (54-criteria/32-point) [5] and simplified (37-criteria/29-point) [9] Selvester QRS scoring systems were evaluated manually by two experienced investigators, blinded to CMR results. Both scoring systems were determined by consideration of 54 and 37 criteria, respectively, based on Q or R wave duration, R or S wave amplitude and R/Q or R/S amplitude ratios. Biochemical measurements of high-sensitivity cardiac troponin T (hs-cTnT) were determined according to the standard protocols of our working group as described in detail previously [10]. A detailed medical history as well as physical examination were carried out during hospitalization and informed consent was obtained from all patients prior study inclusion. The present study protocol was approved by the local research ethics committee in concordance with the Declaration of Helsinki.

### Cardiac magnetic resonance imaging

All CMR scans were performed on a 1.5-T MR imaging unit (AVANTO-scanner, Siemens, Erlangen, Germany) at 3 days (IQR 2–4) as well as 127 days (IQR 121–143) after reperfused STEMI. The detailed imaging and post-processing protocol of our research group was published in detail previously [11]. In brief, volumetric analyses were conducted on short-axis cine images using breath-hold, retrospective ECG-triggered trueFISP bright-blood sequences and for post-processing, standard software (ARGUS, Siemens, Erlangen, Germany) was used. Papillary muscles were attached to the LV volume. 15 min after the application of a 0.2 mmol/kg bolus of contrast agent (Gadovist®, Bayer Vital, Leverkusen, Germany), late gadolinium enhancement (LGE) images were acquired using an ECG-triggered phase-sensitive inversion recovery (PSIR) sequence. LGE extent was determined quantitatively on each slice using IMPAX EE workstation (Agfa HealthCare, Bonn, Germany). “Hyperenhancement” was defined as a threshold of + 5 SD above the signal intensity of remote myocardium in the opposite myocardial segment of the LV [12]. Infarct size (IS) was defined as percentage of LV myocardial mass. Microvascular obstruction (MVO) was characterized as persisting area of “hypoenhancement” within the infarcted myocardium [13]. Experienced readers analyzed all CMR images, blinded to clinical data as well as ECG results.

### Statistical analysis

IBM SPSS Statistics 24.0 (IBM, Armonk, NY, USA) and MedCalc Version 15.8 (Ostend, Belgium) were used for statistical analyses. Continuous variables were expressed as median with corresponding IQR. Categorical variables were presented as absolute numbers with corresponding percentages. Chi-square test was used to assess differences in categorical variables. Differences in continuous variables between two groups were evaluated by Mann–Whitney U-test. Receiver operating characteristic (ROC) curve analysis was applied to evaluate area under the curve (AUC) for the prediction of acute as well as chronic IS and MVO. AUC values were compared according to a method published by DeLong et al. [14]. For multivariable analysis, binary logistic regression analysis was used. A baseline univariable model was created including simplified and complete QRS score as well as hs-cTnT for the prediction of myocardial damage. All parameters showing a *p*-value of < 0.05 in univariable analysis were further included in multivariable analyses. Cut-offs for both QRS scores and large IS were defined by the median of the present cohort. For all analyses, a two-tailed p-value of < 0.05 was considered as statistically significant.

## Results

### Baseline characteristics

We included 201 STEMI patients undergoing pPCI with a median treatment delay of 194 (IQR 128–378) minutes. The mean age of the overall cohort was 56 (IQR 50–67) years. The baseline characteristics are provided in detail by Table 1.

**Table 1:** Patient characteristics

	Total population (*n* = 201)	Simplified QRS < 4 (*n* = 106)	Simplified QRS > 4 (*n* = 95)	*p*-value
Age, years	56 [50–67]	55 [49–63]	59 [52–69]	0.004
Female, n (%)	37 (18)	21 (20)	16 (17)	0.588
Body mass index, kg/m^2^	26 [25–29]	26 [24–28]	27 [25–29]	0.044
Hypertension, n (%)	113 (56)	53 (50)	60 (63)	0.060
Systolic blood pressure, mmHg	130 [114–150]	130 [111–150]	130 [116–150]	0.460
Diastolic blood pressure, mmHg	80 [70–92]	80 [68–90]	80 [71–94]	0.348
Heart rate, min	71 [63–85]	70 [62–83]	74 [64–87]	0.141
Current smoker, n (%)	114 (57)	65 (61)	49 (52)	0.164
Hyperlipidaemia, n (%)	112 (56)	59 (56)	53 (56)	0.958
Diabetes mellitus, n (%)	19 (10)	8 (8)	11 (12)	0.329
Anterior infarct localisation, (%)	92 (46)	35 (33)	57 (60)	<0.001
TIMI flow pre-pPCI				0.158
0	117 (58)	60 (57)	57 (60)	
1	34 (17)	14 (13)	20 (21)	
2	37 (18)	25 (24)	12 (13)	
3	13 (7)	7 (7)	6 (6)	
TIMI flow post-pPCI				0.717
0	1 (1)	1 (1)	0 (0)	
1	3 (2)	1 (1)	2 (2)	
2	21 (10)	11 (10)	10 (11)	
3	176 (88)	93 (88)	83 (87)	
Time from symptom onset to pPCI, min	194 [128–378]	198 [137–373]	189 [123–403]	0.517
Peak hs-cTnT, ng/l	4260 [1931–7214]	3150 [1297–5714]	5708 [2784–9352]	<0.001
IS baseline, % of LVMM	15 [7–24]	13 [5–20]	17 [10–26]	0.001
MVO baseline present	102 (51)	59 (45)	43 (62)	0.003
IS follow-up, % of LVMM	10 [4–15]	8 [2–14]	11 [6–16]	0.003

Abbreviations: *pPCI* Primary percutaneous coronary intervention, *hs-cTnT* high-sensitivity cardiac Troponin T, *IS* Infarct size, *LVMM* Left ventricular myocardial mass, *MVO* Microvascular obstruction

### Clinical associates of QRS scores

Patients with complete QRS scores > median of 12 points were older (*p* = 0.001), showed higher heart rate (*p* = 0.041), higher hs-cTnT levels (*p* < 0.001), larger IS in acute (*p* = 0.004) and chronic (*p* = 0.008) setting and higher presence of MVO (*p* = 0.020).

Patients with simplified QRS scores > median of 4 points were older (p = 0.004), presented with higher body mass index levels (*p* = 0.044), had higher rates of anterior infarct localisation (*p* < 0.001), higher hs-cTnT levels (*p* < 0.001), larger IS in acute (*p* = 0.001) and chronic (*p* = 0.003) setting and higher presence of MVO (*p* = 0.013).

### QRS scoring system and myocardial damage

**Fig. 2:**
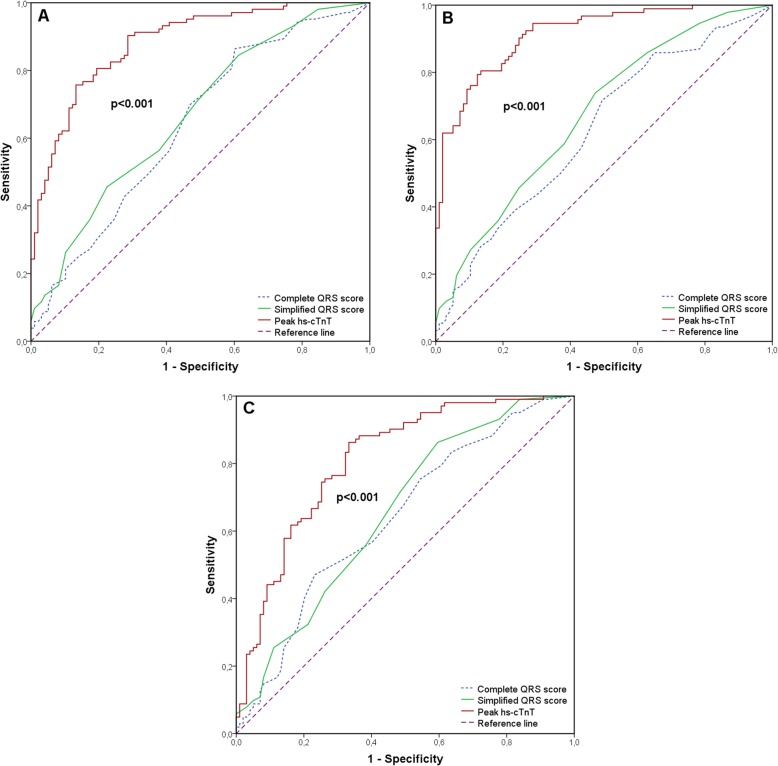
Receiver operating characteristic curves for the prediction of acute IS (> 15% of LVMM) (**a**), chronic IS (> 10% of LVMM) (**b**) and presence of MVO (**c**): Peak hs-cTnT, complete and simplified QRS score. Abbreviations: IS=Infarct size; LVMM = Left ventricular myocardial mass; hs-cTnT = High-sensitivity cardiac troponin T; MVO = Microvascular obstruction

Univariable and multivariable associations between both QRS scoring systems as well as hs-cTnT and myocardial damage are summarized in Table 2. In univariable logistic regression analysis, all three included parameters showed significant association with IS and MVO. In multivariable analysis, including complete, simplified QRS score and peak hs-cTnT, peak hs-cTnT emerged as independent predictor of acute (*p* < 0.001) as well as chronic (p < 0.001) IS and MVO (p < 0.001). AUC values of both QRS scoring systems and hs-cTnT are presented in detail by Table 3. AUC values of complete and simplified QRS score were comparable for the prediction of acute IS (AUC = 0.64, 95% confidence interval (CI) 0.56 to 0.72, *p* = 0.001; AUC = 0.67, 95% CI 0.59 to 0.74, *p* < 0.001, respectively), chronic IS (AUC = 0.63, 95% CI 0.55 to 0.71, *p* = 0.002; AUC = 0.68, 95% CI 0.60 to 0.75, *p* < 0.001, respectively) and MVO (AUC = 0.64, 95% CI 0.56 to 0.72, p = 0.001; AUC = 0.66, 95% CI 0.58 to 0.73, *p* < 0.001, respectively). Peak hs-cTnT resulted in a higher AUC for the prediction of acute (AUC = 0.88, 95% CI 0.83 to 0.93, p < 0.001) and chronic IS (AUC = 0.91, 95% CI 0.88 to 0.95, p < 0.001) as well as for the prediction of MVO (AUC = 0.81, 95% CI 0.75 to 0.87, p < 0.001) (Fig. 2).

**Table 2:** Binary logistic regression analysis for the prediction of IS and MVO

	Univariable analysis		Multivariable analysis	
	**OR (95% CI)**	***p*-value**	**OR (95% CI)**	***p*-value**
*Baseline IS > 15%*				
Complete QRS score	1.07 (1.03 to 1.11)	0.001	–	–
Simplified QRS score	1.22 (1.11 to 1.35)	<0.001	–	–
Peak hs-cTnT	1.00 (1.00 to 1.00)	<0.001	1.00 (1.00 to 1.00)	<0.001
*Chronic IS > 10%*				
Complete QRS score	1.06 (1.02 to 1.11)	0.003	–	–
Simplified QRS score	1.23 (1.11 to 1.36)	<0.001	–	–
Peak hs-cTnT	1.00 (1.00 to 1.00)	<0.001	1.00 (1.00 to 1.00)	<0.001
*MVO present*				
Complete QRS score	1.06 (1.02 to 1.10)	0.003	–	–
Simplified QRS score	1.12 (1.01 to 1.30)	<0.001	–	–
Peak hs-cTnT	1.00 (1.00 to 1.00)	<0.001	1.00 (1.00 to 1.00)	<0.001

Abbreviations: *IS* Infarct size, *MVO* Microvascular obstruction, *OR* Odds ratio, *CI* Confidence interval

**Table 3:** ROC analysis for the prediction of IS and MVO

	AUC	95% CI	*p*-value
*Baseline IS > 15%*			
Complete QRS score	0.64	0.56–0.72	*p* = 0.001
Simplified QRS score	0.67	0.59–0.74	*p* < 0.001
Peak hs-cTnT	0.88	0.84–0.93	*p* < 0.001
Chronic IS > 10%			
Complete QRS score	0.63	0.55–0.71	*p* = 0.002
Simplified QRS score	0.68	0.60–0.75	*p* < 0.001
Peak hs-cTnT	0.91	0.88–0.95	*p* < 0.001
*MVO present*			
Complete QRS score	0.64	0.56–0.72	*p* = 0.001
Simplified QRS score	0.66	0.58–0.73	*p* < 0.001
Peak hs-cTnT	0.81	0.75–0.87	*p* < 0.001

Abbreviations: *ROC* Receiver operating characteristic, *IS* Infarct size, *MVO* Microvascular obstruction, *AUC* Area under the curve, *CI* Confidence interval

## Discussion

This is the first CMR study evaluating a direct comparison of the complete and simplified QRS scoring system as well as in comparison with hs-cTnT for the prediction of myocardial damage in STEMI patients undergoing pPCI. The major findings of the present study can be summarized as follows: Complete and simplified QRS score showed comparable predictive value for acute and chronic IS estimation as well as MVO. However, peak hs-cTnT showed higher predictive value for the assessment of myocardial damage. Taken together, both scoring systems could not show any benefit regarding infarct severity assessment after reperfused STEMI in the era of hs-cTnT.

### QRS scoring system and myocardial damage

The extent of myocardial necrosis is one of the strongest determinants of clinical outcome in STEMI patients [1, 15]. ECG represents a cost-effective, non-invasive and globally available tool allowing an estimation of myocardial damage in the setting of STEMI. Various ECG scoring systems for the assessment of IS have been developed [6, 16, 17]. Selvester et al. developed the QRS score based on knowledge of the normal sequence of ventricular depolarization in dogs and expanded this model to the standard 12-lead ECG and body surface in a simulated male human torso [18, 19]. From this model, criteria for the quantitation of myocardial infarcts from the ECG were developed [20]. Several previous studies have illustrated the usefulness of Selvester QRS scoring system for assessing myocardial necrosis [21, 22]. Before the era of hs-cTnT, positive correlation was found between Selvester QRS score and serum creatine kinase for IS estimation [23, 24]. In the last years, hs-cTnT has been proposed as biomarker of choice for the assessment of myocardial damage in acute STEMI [25]. Tjandrawidjaja et al. described significant relation between peak troponin T and complete QRS score in a large STEMI cohort [26]. In line with the latter study, we also found significant association between both QRS scoring systems and hs-cTnT. Nowadays CMR represents the gold standard for estimating IS in vivo [1]. Concordant with our study, only moderate correlation was found between CMR derived IS and complete Selvester QRS score [21, 27].

Besides IS, microvascular injury emerged as strong predictor of adverse cardiovascular events after STEMI [15, 28, 29]. Previous data reported a rate of MVO up to 55% in patients after revascularized STEMI [28]. This is consistent with the present data, naming a rate of MVO in 51% of our study population. Data concerning the relationship between Selvester QRS score and MVO are limited [21]. Watanabe et al. reported in a small cohort of 62 patients with acute myocardial infarction significant correlation between simplified QRS score and CMR determined MVO volume [30]. Anyway, previous studies did not directly compare associations between complete and simplified QRS score with microvascular injury. The present study provides deeper insights as we investigated a direct comparison of both QRS scores regarding MVO. In this comparison, both QRS scoring systems showed comparable predictive value for estimating MVO. These results confirm the theory of the association between high QRS scores with irreversible myocardial damage [30]. However, concordant with our data, hs-cTnT still represents one of the best parameters evaluating microvascular injury [31, 32].

### Limitations

Limitations of the present study have to be declared. Firstly, we included relatively stable STEMI patients with Killip class < III in order to perform high-quality CMR imaging. Therefore, these data may not be generalizable for patients in worse clinical condition. Nevertheless, the majority of STEMI patients represent with Killip class < III [33]. Secondly, Selvester QRS scores were evaluated at a single time point, and therefore their predictive value might be different if measured on other time points. Thirdly, T2-weighted edema was not included in the present study mostly due to the limited validity of this imaging sequence [34, 35].

## Conclusions

Complete and simplified Selvester QRS score showed comparable predictive value for the assessment of CMR derived IS and MVO in reperfused STEMI. However, compared to peak hs-cTnT, the clinical reference method for the assessment of myocardial damage, both QRS scoring systems revealed inferior predictive value.

## Data Availability

The raw data of this study will not be shared publically because they will be applied for further researches of this series, but authors do agree that the data can be shared individually if requested.

## Abbreviations

AUC: Area under the curve
CI: Confidence interval
CMR: Cardiac magnetic resonance
ECG: Electrocardiography
hs-cTnT: High-sensitivity cardiac troponin T
IQR: Interquartile range
IS: Infarct size
LGE: Late gadolinium enhancement
LV: Left ventricular
LVMM: Left ventricular myocardial mass
MVO: Microvascular obstruction
pPCI: Primary percutaneous coronary intervention
PSIR: Phase sensitive inversion recovery
ROC: Receiver operator characteristics
SD: Standard deviation
STEMI: ST-segment elevation myocardial infarction
